# Are physician assistant and patient airway assessments reliable compared to anesthesiologist assessments in detecting difficult airways in general surgical patients?

**DOI:** 10.1186/s13741-017-0077-0

**Published:** 2017-11-22

**Authors:** Erin Payne, Jacqueline Ragheb, Elizabeth S. Jewell, Betsy P. Huang, Angela M. Bailey, Laura M. Fritsch, Milo Engoren

**Affiliations:** Department of Anesthesiology, University of Michigan, 4383 Cardiovascular Center, 1500 E. Medical Center Drive, Ann Arbor, MI 48109-5861 USA

**Keywords:** Airway assessment, Mallampati, Instrument, Anesthesiologist

## Abstract

**Background:**

Airway management remains one of the most important responsibilities of anesthesiologists. Prediction of difficult airway allows time for proper selection of equipment, technique, and personnel experienced in managing patients with difficult airway. Face to face preoperative anesthesia interviews are difficult to conduct as they necessitate patients traveling to the clinics, and, in practice, are usually conducted in the morning of the procedure by the anesthesiologist, when identification of predictors of difficult intubation may lead to schedule delays or case cancelations. We hypothesized that an airway assessment tool could be used by patients or physician assistants to accurately assess their airways.

**Methods:**

We administered an airway assessment tool, which had been constructed in consultation with a psychometrician and revised after non-medical layperson feedback, to 215 patients presenting to the preoperative clinic for evaluation. Separately, patients had the airway exam performed by a physician assistant and an anesthesiologist. Agreement was compared using kappa.

**Results:**

We found good agreement between observers only on “can you put three fingers in your mouth?” (three-way kappa = .733, *p* < 0.001) and poor agreement on Mallampati classification (three-way kappa = .195, *p* < 0.001) and “Can you fit three fingers between your chin and your Adam’s Apple?” (three-way kappa = .216, *p* < 0.001). The agreements for the other questions were mostly fair. Agreements between patients and anesthesiologists were similar to those between physician assistants and anesthesiologists.

**Conclusions:**

Neither the patients’ self-assessments nor the physician assistants’ assessments were adequate to substitute for the anesthesiologists’ airway assessments.

## Background

Airway management remains one of the most important responsibilities of anesthesiologists (2003). Prediction of difficult airway allows time for proper selection of equipment, technique, and personnel experienced in managing patients with difficult airway. It is important to identify this group of patients as unanticipated difficult airway may result in catastrophic outcomes such as brain damage or death (Cook et al. 2011). Closed claim analysis has found that the vast majority (85%) of airway-related events involve brain damage or death (Cook et al. 2010).

In major multi-site surgical facilities, expertise and equipment to accommodate patients who have difficult airway may not be available on all surgical sites. This may lead to procedure cancelations or deferrals on the morning of surgery. Late deferrals are a major cause of inefficient use of operating-room time and waste of resources and are unsatisfactory for both patients and surgical teams (Kumar and Gandhi 2012; Garg et al. 2009).

Although anesthesia preoperative assessment clinics are widely available in different institutions to identify preoperative morbidities and optimize patients for surgery, this is mostly conducted by screening patients’ medical records and phone interviews. Face to face preoperative anesthesia interviews are difficult to conduct as they necessitate patients traveling to the clinics, and, in practice, are usually conducted in the morning of the procedure by the anesthesiologist.

Patient self-assessment tools and questionnaires have been used to obtain an overall health assessment, to explore the effects of any medical problems on the everyday activity of the patients, to identify needs of medical interventions, and to guide the necessity for elective and emergency hospital admission (Wasson et al. 1999; Miyamichi et al. 2012). These assessment tools, used by patients and validated by clinicians, have been shown to identify medical conditions for treatment and reduce overcrowding of health facilities, including unnecessary clinic visits and hospital admissions.

We hypothesized that a patient assessment tool used by the patient or by a physician assistant would be useful in identifying patients with difficult airway and hence ensuring their allocation and management to a well-equipped and staffed facility. Validation of such a tool will facilitate airway examination in the preoperative setting. This will minimize same-day cancelation or deferral in surgical facilities lacking tools and equipment to deal with this patient population.

## Methods

**Fig. 1:**
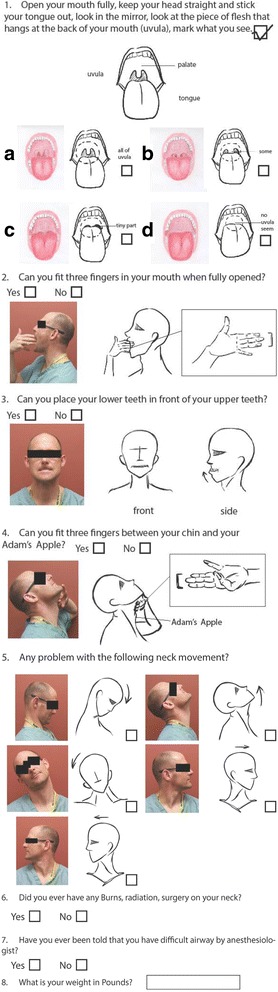
Patient assessment tool

After consultation with a psychometrician, we designed a patient assessment tool (Fig. 1) with illustrations of the major difficult airway predictors and key questions to identify patients who may have a difficult airway (Sim and Wright 2005; L’Hermite et al. 2009). The tool was then shown to other anesthesiologists, who provided feedback, and the tool was then adjusted to meet their suggestions. Next, the tool was shown to non-medical laypersons for their suggestions and anesthesiologist observation of their performance using the form. Additional changes were made. This revised tool was then shown to another group of non-medical laypersons, who appeared to have good performance of an airway self-assessment and which elicited no further suggestions.

After Institutional Review Board approval and subject informed consent, a non-consecutive sample of 215 patients presenting to the perioperative clinic for history and physical examination between July 2015 and May 2016 was asked to complete the airway tool (Fig. 1) during their visit. They were chosen based on the availability of the consenters, PAs, and anesthesiologists. A subsequent independent assessment using the same tool was performed by four clinic physician assistants (PA) and separately by an anesthesiologist. The PAs were selected based on willingness to participate and provided informed consent. Each PA had various levels of experience working in our clinic, but each was given training on airway examination by an anesthesiologist prior to this study as they do not routinely include an airway exam in their surgical history and physical examination.

Adult English-speaking patients undergoing non-cardiac surgical procedures who presented to the surgical clinic on the days one of the study’s authors (EP, JR) was present were approached for consent. Patients were excluded if they were < 18 years of age, do not speak English, or were unable to consent due to guardianship. The tool and written instructions (Fig. 1) were given to the patients by the non-medical research assistant obtaining consent. The study was conducted using STROBE criteria for cohort studies (http://www.strobe-statement.org/index.php?id=available-checklists); accessed November 6, 2017.

### A priori power analysis

Assuming that the average proportion of positive ratings on a dichotomous question is 0.7, that the raters are unbiased, that the two-tailed null value is 0.5, and that the pairwise kappa we wish to detect is 0.7, we would need 173 subjects (Sim and Wright 2005). To correct for possible systemic biases, we planned to recruit 215 subjects.

### Statistical analysis

Patients’ demographics were described using frequency and proportion for categorical data and means and standard deviations for continuous data. A comparison between the results of the three assessments made by patient, PA, and anesthesiologist was conducted on each of the five airway measures, using kappa: pairwise (Cohen) comparisons (PA-patient, PA-anesthesiologist, patient-anesthesiologist). We also computed a group (Fleiss) kappa for the three-way agreement. kappa ≥ 0.7 was considered good agreement.

## Results

Of the 215 patients, 122 (57%) were female. Most (*n* = 102, 47%) had a high school education, and 68 (32%) had college education, while only 45 (21%) had less than a high school education. Patients were (mean ± standard deviation) 59 ± 15 years old and weighed 86 ± 50 kg, with a maximum weight of 185 kg.

We found good agreement between observers only on question 2 “can you put three fingers in your mouth?” and poor agreement on question 1—Mallampati classification—and question 4 “Can you fit three fingers between your chin and your Adam’s Apple?”. The agreements for the other questions were mostly fair (Table 1).

**Table 1:** Agreement (kappa) between the groups

Item	Pat-PA		PA-Anes		Pat-Anes		Pat-PA-Anes	
	Kappa	*p*	Kappa	*p*	Kappa	*p*	Kappa	*p*
1	.192	< .001	.202	< .001	.216	< .001	.216	< .001
2	.807	< .001	.681	< .001	.723	< .001	.733	< .001
3	.542	< .001	.509	< .001	.527	< .001	.523	< .001
4	.205	.003	.179	.011	.205	.004	.195	< .001
5 Down	.659	< .001	.659	< .001	.590	< .001	.634	< .001
5 Up	.486	< .001	.535	< .001	.360	< .001	.456	< .001
5 Tilt	.152	.002	.348	< .001	.101	.051	.212	< .001
5 Left	.485	< .001	.260	< .001	.260	< .001	.328	< .001
5 Right	.534	< .001	.658	< .001	.654	< .001	.619	< .001

Item 1 is Mallampati classification, 2 is “Can you fit three fingers in your mouth when fully opened?,” 3 is “Can you place your lower teeth in front of your upper teeth?,” 4 is “Can you fit three fingers between your chin and your Adam’s apple?,” and 5 is neck motion and position (Fig. 1). *P* is the probability that kappa differs from zero by chance alone

*Pat* patient, *PA* physician assistant, *Anes* anesthesiologist

**Fig. 2:**
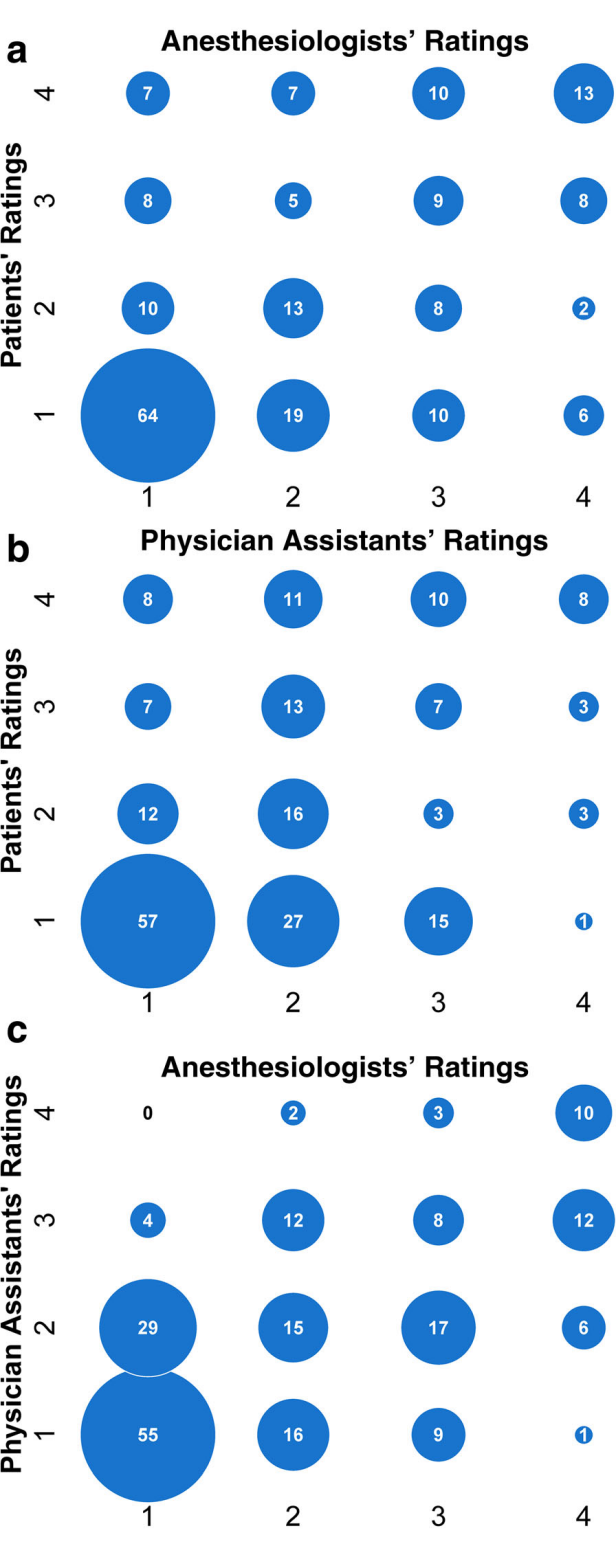
Bubble plots showing the agreement between **a** patients’ and anesthesiologists’ assessments, **b** patients’ and physician assistants’ assessment, and **c** and physician assistants’ and anesthesiologists’ assessment of Mallampati class. One patient was examined by both the anesthesiologist and the physician assistant, but not by self. Twelve patients were missing both the anesthesiologist and the physician assistant assessments. Three patients were missing only the anesthesiologist’s assessment, and one was missing only the physician assistant’s assessment

Notably, we also found that the agreements between the patients’ assessments and the anesthesiologists’ assessments were very similar to the agreements between the PA’s assessments and the anesthesiologists’ assessments. While patients and anesthesiologists agreed on the Mallampati class half the time (50%), there was no consistent bias between grader and different scores. Patients were as likely to overgrade (*n* = 47) as undergrade (*n* = 53), *p* = .564. Similarly, PA’s were as likely to overgrade (*n* = 50) as undergrade (*n* = 61) compared to anesthesiologists, *p* = .264. Of the patients whom the anesthesiologist graded the airway as Mallampati IV, patients only agreed 45% of the time and PAs only 34% (Fig. 2).

## Discussion

In this study, we evaluated the interrater agreement using an airway assessment tool between patients, PAs, and anesthesiologists in a preoperative clinic setting. We hypothesized that using this airway assessment tool (Fig. 1), patients would be able to reliably and accurately assess their airway compared to the anesthesiologist and thus would be useful in identifying patients with difficult airways and ensuring that this patient population is managed in the appropriate facility with sufficient resources. We included all risk predictors for a difficult airway that are routinely used and documented at the University of Michigan for an airway exam in the design of our assessment tool: Mallampati classification, inter-incisor gap, thyromental distance, and cervical flexion and extension (L’Hermite et al. 2009; Khan et al. 2009).

The Joint Commission’s standards for a hospital of surgery center to be accredited require a pre-procedure airway assessment; however, it does not specify the required elements of the assessment (https://www.jointcommission.org/standards_information/jcfaqdetails.aspx?StandardsFaqId=869&Programid=46; accessed November 6, 2017). As the Joint Commission also has a “comparable care” mandate, many healthcare facilities have interpreted these standards to require either the non-anesthesiologist performing the procedure with conscious sedation or a designee, usually the procedure room registered nurse, to perform an airway assessment similar to that performed by an anesthesiologist (https://www.patientsafety.va.gov/professionals/onthejob/sedation.asp; accessed November 6, 2017). Guidelines for the performance of the airway assessment by non-anesthesiologists have been promulgated by the American Society of Anesthesiologists (American Society of Anesthesiologists Task Force on Sedation and Analgesia by Non-Anesthesiologists 2002). However, there are scant studies determining how well non-anesthesiologists perform components of an airway assessment. Kandray et al. evaluating Mallapati classification found that one dentist and 21 dental hygiene students examining a sample of 234 patients agreed 77% (95% confidence interval = 72, 82%) of the time with kappa = 0.54 (0.42, 0.64), which is higher than the kappas found for Mallampati classification (Kandray et al. 2013). A study at a university medical center found that the agreement on Mallampati classification between gastroenterologists and anesthesiologists was no better than chance: kappa = 0.103 (− 0.0126, 0.219), when gastroenterologists were compared to other gastroenterologists, the agreement was similarly poor: kappa = 0.120 (− 0.0211 to 0.260), and when compared among themselves doing the examination twice, gastroenterolgists had only moderate agreement between their first and second exam of the same patient: kappa = 0.420 (0.119, 0.722) (Lopez et al. 2014). Even the agreement among anesthesiologists is variable. A Danish study calculated kappa between two anesthesiology specialists and two anesthesiology residents on measures of an airway assessment consisting of the measurement of the mouth opening, the thyromental distance, the ability to protrude the mandible, and an evaluation of the Mallampati class and head and neck mobility in 136 patients scheduled for elective surgery. The kappa for residents was 0.28 (0.05, 0.61) and specialists was 0.39 (0.06, 0.72) for neck mobility and 0.41 (0.30, 0.52) and 0.80 (0.65, 0.95), respectively, for Mallampati classification (Rosenstock et al. 2005). Our findings are in agreement with these studies, but are novel in assessing patient self-assessment and including more components of an airway exam.

Our results showed that for most components of the airway assessment, there is less than good agreement between patients and experts, making self-assessment unreliable to identify potential difficult airways. In particular, patients were only able to identify a Mallampati IV airway, as rated by an anesthesiologist, 45% of the time (Fig. 2). These findings suggest that this tool cannot be used by patients to evaluate their airways. As it may be burdensome for some patients to physically visit an anesthesiologist for the important airway assessment, other less burdensome methods should be investigated, e.g., cellular phone videoconferencing may permit a remote anesthesiologist to conduct the airway assessment, but would need to be first studied.

Operationally, the direction of disagreement between patient and anesthesiologist or PA and anesthesiologist may be important. A patient or PA who scores a low Mallampati score and normal mouth opening, thyromental distance, and cervical mobility but has a Mallampati IV airway and decreased mouth opening, thyromental distance, and cervical mobility as assessed by the anesthesiologist and hence requires an unanticipated awake fiber-optic intubation may disrupt the scheduling and workflow at the facility or lead to a postponed case. Conversely, if the patient or PA assesses Mallampati IV and other markers of a difficult intubation and the anesthesiologist disagrees, there may be no disruption to the facility—only the fiber-optic scope is put away unused. Importantly, we found no pattern between over- and under-scoring the components of the airway assessment.

We also found disappointingly similar poor agreement with the same difficult airway predictors comparing PAs and anesthesiologists. This could reflect the difference in training or experience among providers. It could also reflect lack of standardization of airway assessment descriptors, e.g., two raters may see the identical view, but not have a standard definition of finger size in rating inter-incisor or thyromental distance, leading to different ratings. This has implications because many mid-level providers are often tasked with airway assessment in various clinical settings (https://www.patientsafety.va.gov/professionals/onthejob/sedation.asp; accessed November 6, 2017). This can lead to inappropriate patient allocation to centers with insufficient resources in managing difficult airways or the patient inappropriately receiving conscious sedation by the surgeon instead of monitored anesthesia care by the anesthesiologist. Given the importance of non-anesthesiologists performing airway assessments, research is needed on how best to improve their airway assessments.

In contrast to other studies demonstrating interrater variability in airway assessment, our study is the first to include patients using a self-assessment tool (Kandray et al. 2013; Rosenstock et al. 2005). The main strength of our study is the design and validation of the tool with a psychometrician and subsequent testing as well as revision based on feedback from both anesthesiologists and non-medical laypersons. There are several limitations to our study. First, we did not determine the agreement between the two anesthesiologists in this study. Disagreements between anesthesiologists may suggest that the airway assessment is not sufficiently standardized, either in the definitions or in the performance. We did not assess which provider (PA or anesthesiologist) or patient best assessed the airway for a difficult intubation as we did not follow patients through to intubation. Another limitation is the limited amount of training and experience given to the PAs prior to this study. Patients came from a small geographic area with a relatively homogenous population and our results may not be generalizable to different patient populations. Finally, we did not debrief the patients after the self-conducted airway assessment to determine the reasons why patients ascribed a particular value to each component of the assessment.

## Conclusions

We found that agreements between patients and anesthesiologists on different components of an airway examination vary by component but the overall is poor to good. We also found that the agreements between PAs and anesthesiologists on the same airway examination also vary by component but the overall is poor to good. This suggests that neither the patient’s self-assessment nor the PA’s assessment of an airway can reliably and accurately replace the anesthesiologist’s examination.

## Abbreviations

PA: Physician assistant
